# Awake Tracheostomy in the Sitting Position for a Predicted Difficult Airway in Ankylosing Spondylitis With Inhalation Injury: A Case Report

**DOI:** 10.1155/cria/7792815

**Published:** 2026-09-20

**Authors:** Van Quynh Nguyen, Quang Thao Le, Thi Van Nguyen

**Affiliations:** ^1^ Department of Anesthesia, Le Huu Trac National Burn Hospital, Hanoi, Vietnam; ^2^ Intensive Care Unit, Le Huu Trac National Burn Hospital, Hanoi, Vietnam; ^3^ Department of Paraclinical Medicine, Le Huu Trac National Burn Hospital, Hanoi, Vietnam

**Keywords:** ankylosing spondylitis, awake tracheostomy, difficult airway, inhalation injury, sitting position

## Abstract

**Background:**

Airway management in patients with inhalation injury is particularly challenging because of rapidly progressive airway edema. This difficulty is further compounded in patients with ankylosing spondylitis, in whom cervical spine rigidity and deformity severely restrict neck mobility and may prevent standard positioning for airway interventions. In such situations, conventional airway management techniques may fail, necessitating alternative strategies.

**Case Presentation:**

We report the case of a 48‐year‐old man with inhalation injury following a flame burn who also had long‐standing ankylosing spondylitis with marked cervicothoracic deformity. The patient developed progressive respiratory failure with impending airway obstruction. Emergency endotracheal intubation was attempted twice using a conventional Macintosh direct laryngoscope but was unsuccessful because of severe positional limitations, restricted cervical mobility, and progressive airway edema.

**Management and Outcome:**

An emergency awake tracheostomy was performed in the sitting position at approximately 90° under local anesthesia with light sedation while spontaneous ventilation was maintained. Despite significant challenges related to anatomical deformity and soft‐tissue edema, the procedure was successfully completed within approximately 10 min. Oxygen saturation improved immediately from approximately 85% to 99%–100%, and no immediate procedure‐related complications were observed. Bronchoscopy performed after airway stabilization demonstrated Grade II inhalation injury according to the abbreviated injury score. The patient subsequently remained mechanically ventilated through the tracheostomy for 22 days and underwent two burn wound excision and autologous skin grafting procedures. He later developed sepsis progressing to septic shock and multiple‐organ failure and died on Day 22 after tracheostomy.

**Conclusion:**

Emergency awake tracheostomy in the sitting position may be an effective option for securing the airway when conventional airway techniques are unsuccessful and supine positioning is not feasible. This case highlights the importance of individualized airway management, preservation of spontaneous ventilation, and readiness to transition promptly to a surgical airway in patients with severe anatomical and acute airway challenges.

## 1. Introduction

Inhalation injury is a severe burn‐related complication associated with increased mortality and may rapidly progress to respiratory failure if not recognized and managed promptly. Thermal and chemical injuries to the upper airway, particularly the supraglottic structures, together with the ensuing inflammatory response, can lead to progressive airway edema and significantly increase the difficulty of airway management, especially during the early postburn period [1].

Difficult airway management remains a major challenge in anesthetic and critical care practice, particularly in emergency situations encountered in the intensive care unit. When endotracheal intubation fails, alternative airway strategies must be implemented promptly to maintain adequate ventilation and oxygenation. In situations where conventional airway techniques are unable to secure the airway, timely selection of an appropriate alternative approach is crucial to prevent severe hypoxic complications [2].

In patients with ankylosing spondylitis, progressive rigidity and deformity of the cervical and thoracic spine can severely restrict neck mobility, making endotracheal intubation and other airway interventions particularly challenging [3].

Tracheostomy is conventionally performed in the supine position with neck extension to facilitate exposure of the trachea. However, this position may be impossible in certain patients because of anatomical limitations, necessitating alternative approaches. Reports describing emergency tracheostomy performed in nonconventional positions, including the sitting position, remain scarce in the literature.

We report a case of inhalation injury in a patient with ankylosing spondylitis who presented with a difficult airway and failed endotracheal intubation and was successfully managed with awake tracheostomy performed in the sitting position.

## 2. Case Presentation

A 48‐year‐old man sustained gasoline flame burns at approximately 9:00 p.m. on October 14, 2025, while smoking. After the injury, he received initial first aid and was transferred to a local hospital before being referred to the National Burn Hospital approximately 6 h after the burn injury.

On admission, the patient was conscious and communicative. He was breathing spontaneously at a respiratory rate of 22 breaths/min, with an oxygen saturation of 96% on room air, a heart rate of 115 beats/min, and a blood pressure of 125/80 mmHg. Physical examination revealed singed scalp hair, eyebrows, eyelashes, and nasal hair, together with facial and cervical edema and swollen lips. Burn injuries involved 27% of the total body surface area, including 8% full‐thickness burns, affecting the head, face, neck, chest, back, and both upper limbs. These findings were accompanied by clinical signs suggestive of inhalation injury.

**FIGURE 1:**
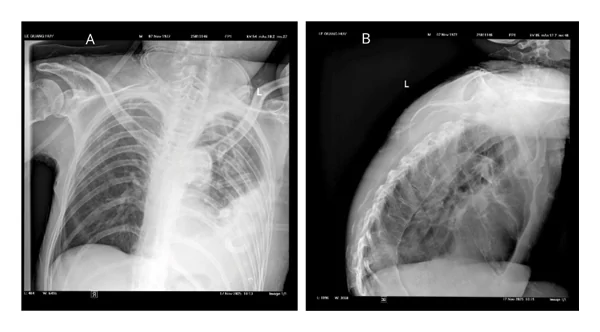
Anteroposterior (A) and lateral (B) radiographs of the cervical and thoracic spine demonstrating a fixed kyphotic cervicothoracic deformity secondary to ankylosing spondylitis.

The patient had a longstanding history of ankylosing spondylitis with marked spinal deformity and severely restricted cervical mobility. Cervical and thoracic spine radiographs demonstrated a fixed kyphotic cervicothoracic deformity with severe limitation of neck extension, preventing the patient from assuming the supine position (Figure 1).

Airway assessment indicated a very high risk of difficult endotracheal intubation because of progressive edema involving the head, face, and neck, combined with severely restricted cervical spine mobility and the inability to position the patient supine. During hospitalization, the patient received fluid resuscitation, supplemental oxygen therapy, inhalation treatment, analgesia, and close monitoring of vital signs. During the clinical course, the patient developed upper gastrointestinal bleeding accompanied by severe metabolic acidosis and was managed with aggressive resuscitative treatment.

Approximately 12 h after the burn injury, the patient’s respiratory status deteriorated, with agitation, restlessness, increased airway secretions, progressive upper‐airway edema, and a decrease in SpO_2_ to approximately 90%. Emergency endotracheal intubation was attempted twice using a conventional Macintosh direct laryngoscope, with the two attempts approximately 1‐2 min apart. The glottis could not be visualized because of the fixed cervicothoracic deformity, severely restricted cervical mobility, inability to achieve conventional intubating positioning, and progressive upper‐airway edema. A formal Cormack–Lehane grade was not documented during these emergency attempts.

A fiberoptic bronchoscope was available, and awake fiberoptic intubation was considered. However, the patient’s airway edema was progressing rapidly and oxygen saturation continued to decrease, increasing the risk of severe hypoxemia and complete loss of the airway if definitive airway control was further delayed. Immediately after the second unsuccessful direct laryngoscopy attempt, the team, therefore, decided not to pursue further tracheal intubation attempts and proceeded to emergency awake tracheostomy while spontaneous ventilation was maintained.

**FIGURE 2:**
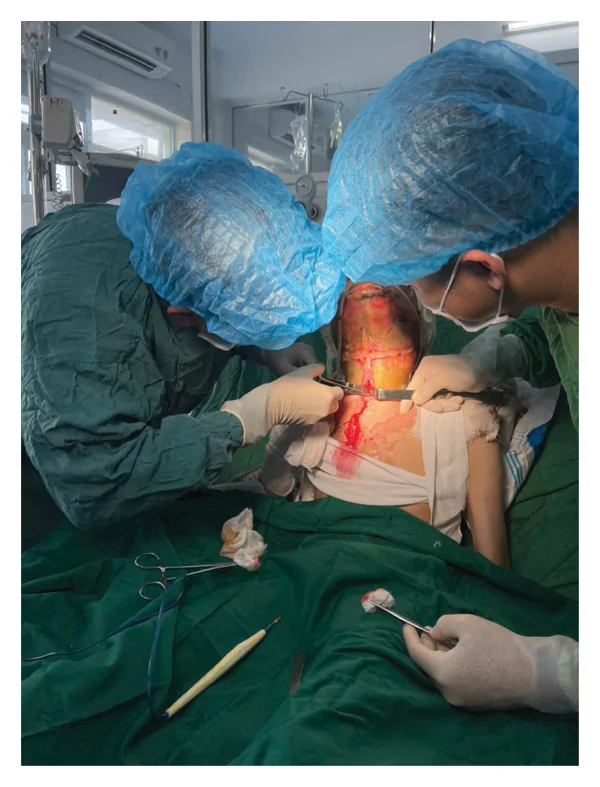
Emergency awake tracheostomy performed in the fully sitting position at approximately 90° because supine positioning was not feasible.

At the time of this decision, the patient remained unable to tolerate the supine position because of his fixed cervicothoracic deformity. Therefore, the tracheostomy was undertaken with the patient in a fully sitting position at approximately 90° (Figure 2). The procedural details are described below.

## 3. Tracheostomy Technique

At the time of the procedure, the patient was in respiratory distress, with agitation, tachypnea, and audible wheezing. Oxygen saturation was approximately 85% despite supplemental oxygen delivered via a nasal cannula at 10 L/min. Spontaneous ventilation was maintained throughout the procedure, and no additional oxygen‐delivery device was used before establishment of the surgical airway. Continuous pulse oximetry and intermittent noninvasive blood pressure monitoring were maintained. ECG monitoring could not be reliably applied because of extensive deep burns involving the anterior chest. Airway secretions were suctioned frequently to maintain airway patency.

Because of the fixed cervicothoracic deformity and inability to tolerate the supine position, the patient remained in a fully upright sitting position at approximately 90°. A supporting pad was placed at the cervical spine, and an assistant manually stabilized the head and neck to minimize movement throughout the procedure. This position allowed the patient to maintain spontaneous ventilation while providing surgical access to the anterior neck.

Local anesthesia was achieved by infiltration of 4 mL of 1% lidocaine (total dose, 40 mg) at the tracheostomy site. Light sedation was provided with 2.5 mg of intravenous midazolam. No formal sedation scale, such as the Ramsay Sedation Scale or Richmond Agitation‐Sedation Scale, was used; sedation was assessed clinically by maintaining patient responsiveness and spontaneous ventilation.

The procedure was performed by an intensive care physician with 15 years of experience, including extensive experience in tracheostomy and difficult airway management in critically ill patients with burns and inhalation injury. A conventional open tracheostomy technique was used, with a high tracheal opening at the level of the first to third tracheal rings. The procedure was technically challenging because deep burns involving the anterior neck made anatomical landmarks difficult to identify. Subcutaneous edema and fluid leakage further limited exposure of the trachea.

A contingency plan was prepared in case the surgical airway could not be successfully established. The priorities were to maintain spontaneous ventilation and oxygenation, reassess the surgical opening, suction blood, or secretions obscuring the operative field, reexpose the tracheal lumen, and reattempt placement of the tracheostomy tube while minimizing interruption of spontaneous breathing.

Despite these difficulties, the trachea was successfully accessed and a cuffed 7.5 mm tracheostomy tube was inserted. The procedure was completed within approximately 10 min. Correct tube placement was confirmed by bilateral chest auscultation and capnography. The patient was immediately connected to mechanical ventilation in the Burn ICU using volume‐controlled ventilation (VCV) with a lung‐protective strategy and a tidal volume of 4–8 mL/kg of predicted body weight. Oxygen saturation rapidly improved from approximately 85% to 99%–100%, and the patient became less agitated and clinically more stable. No immediate procedure‐related complications, including cardiac arrest, apnea, or significant bleeding, were observed.

**FIGURE 3:**
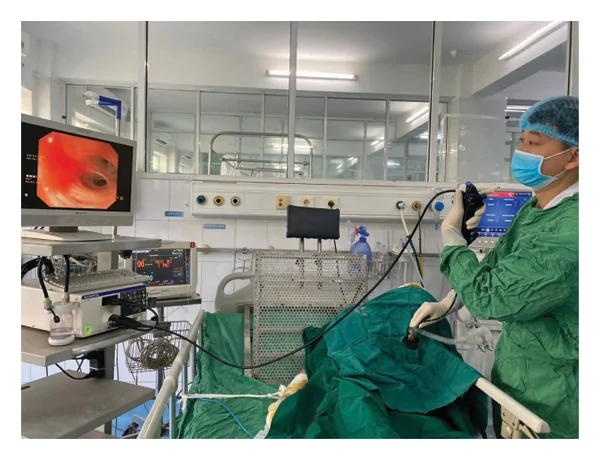
Bronchoscopic findings demonstrating Grade II inhalation injury according to the abbreviated injury score.

After the airway had been secured and the patient’s respiratory status stabilized, flexible bronchoscopy was performed immediately through the tracheostomy to assess the airway injury. Bronchoscopic examination demonstrated Grade II inhalation injury according to the abbreviated injury score (AIS) (Figure 3).

### 3.1. Subsequent Clinical Course and Outcome

Following successful tracheostomy, the patient remained mechanically ventilated through the tracheostomy for 22 days until his death. During this period, he continued to receive intensive burn care and underwent two surgical procedures for burn wound excision and autologous skin grafting.

Despite ongoing intensive treatment, the patient subsequently developed sepsis, which progressed to septic shock and multiple‐organ failure. Because he remained critically ill and dependent on mechanical ventilation, decannulation was not possible, and the tracheostomy tube remained in place throughout the remainder of his clinical course. The patient ultimately died on Day 22 after tracheostomy from septic shock and multiple‐organ failure.

## 4. Discussion

### 4.1. Ankylosing Spondylitis and Difficult Airway

Ankylosing spondylitis is a well‐recognized challenge in airway management because progressive ankylosis of the cervical spine can result in marked limitation of neck extension, reduced mouth opening, and fixation of the atlantoaxial joints [4]. In patients with advanced disease, cervical spine deformity and ankylosis may make supine positioning for direct laryngoscopy difficult or impossible. In addition, restricted mobility of the cervical spine and temporomandibular joints can substantially reduce mouth opening, thereby limiting direct laryngoscopic visualization and increasing the risk of difficult endotracheal intubation [3].

Attempts to force patients with ankylosing spondylitis into a standard airway position carry a considerable risk of neurologic injury, as the ankylosed cervical spine may become susceptible to fracture even after minor trauma. Maneuvers that mobilize the cervical spine, particularly attempts to achieve the sniffing position, may increase the risk of spinal cord injury [3, 5]. These anatomical limitations require early recognition and an appropriate airway management plan, particularly when combined with acute conditions such as inhalation injury, which can further aggravate airway narrowing and obstruction.

Airway assessment in patients with ankylosing spondylitis should be comprehensive and include evaluation of cervical spine mobility, mouth opening, and other predictors of difficult intubation. In patients with severe cervical deformity, awake tracheal intubation, particularly flexible bronchoscopic intubation, is generally an important option when feasible because it allows preservation of spontaneous ventilation and minimizes cervical spine manipulation [3]. However, the optimal strategy must be individualized according to the urgency of airway compromise, anatomical limitations, available techniques, and the likelihood of successful intubation.

### 4.2. Inhalation Injury: A Narrow Window for Airway Intervention

Inhalation injury following thermal burns is characterized by rapidly progressive airway edema. Although initial clinical signs may be subtle, the condition can quickly progress to significant airway obstruction, necessitating close monitoring and timely airway intervention [6]. The underlying pathophysiology involves direct thermal injury to the airway mucosa, combined with the effects of toxic combustion products and a local inflammatory response, leading to edema and structural changes within the airway wall. These changes contribute to airway narrowing and impaired respiratory function [6, 7].

Clinical findings suggestive of inhalation injury include facial burns, singed nasal hair, carbonaceous sputum, hoarseness, stridor, and respiratory distress. However, the absence of these signs does not exclude airway injury, and flexible laryngoscopy may identify early airway edema before clinical deterioration becomes apparent [1].

Delayed recognition and airway intervention in burn patients may increase the risk of difficult endotracheal intubation, as clinical manifestations often develop only after airway narrowing has become significant. This may lead to airway management–related complications, including hypoxemia and its associated consequences [8]. Therefore, the decision to secure the airway should be considered early, particularly during the initial postinjury period, as progressive edema may render subsequent airway interventions increasingly difficult and increase the risk of progression from an anticipated difficult airway to an airway that cannot be safely controlled.

In the present case, the initial clinical findings were concerning for inhalation injury, but the patient remained spontaneously breathing with an SpO_2_ of 96% on room air at admission. Over the subsequent hours, progressive airway edema, increased secretions, agitation, and hypoxemia developed. Bronchoscopy performed after definitive airway control confirmed Grade II inhalation injury according to the AIS. This clinical progression illustrates how the window for securing the airway may narrow rapidly in patients with inhalation injury, particularly when a preexisting anatomical abnormality further limits conventional airway management.

### 4.3. The Role of Awake Tracheostomy

Awake tracheostomy performed under local anesthesia offers several advantages in patients with an anticipated difficult airway and a high risk of progressive respiratory compromise, particularly when conventional airway management techniques are likely to fail. Awake airway management strategies are recommended in such situations to minimize the risk of adverse events, including the “cannot intubate, cannot ventilate” scenario, which may result in severe hypoxemia and its associated complications [9].

In burn patients with inhalation injury and a potentially difficult airway, awake airway management techniques are recommended to preserve spontaneous ventilation and reduce the risk of losing airway control during intervention [10]. When endotracheal intubation is not feasible, surgical airway techniques, including tracheostomy, may be indicated to ensure effective ventilation and oxygenation [11]. Although technically demanding, awake tracheostomy may serve as an effective alternative airway strategy in emergency situations when conventional techniques are not feasible or have failed.

A focused literature review identified several reports relevant to awake airway management in patients who could not tolerate conventional positioning, but none closely reproduced the full combination of features present in our case.

Awake tracheostomy has previously been reported in patients with impending airway obstruction who could not tolerate the supine position. Ahuja et al. described a 10‐year‐old child with a retropharyngeal abscess who underwent successful awake tracheostomy while sitting in the most comfortable position to avoid respiratory distress; however, that patient did not have ankylosing spondylitis or inhalation injury [12].

In patients with severe ankylosing spondylitis, published reports predominantly describe difficult or alternative tracheal intubation strategies rather than emergency awake tracheostomy. Brezic et al. reported a patient with severe cervical deformity in whom direct laryngoscopy, videolaryngoscopy, and fiberoptic bronchoscope‐assisted intubation were unsuccessful, and surgical tracheostomy was considered unfeasible because of the neck deformity [13].

Tracheostomy itself may also be technically challenging in advanced ankylosing spondylitis. Aggarwal et al. described a patient with a fixed flexed neck in whom surgical tracheostomy required extensive preprocedural planning and specialized positioning and surgical exposure because access to the anterior neck was severely restricted [14].

Awake airway management in the upright position has also been described in other clinical settings. Ayuningtyas et al. reported successful awake face‐to‐face videolaryngoscopic intubation in a patient with severe maxillofacial trauma who could not tolerate the supine position because of ongoing bleeding and aspiration risk [15].

The novelty of the present case, therefore, lies in the combined presence of advanced ankylosing spondylitis with fixed cervicothoracic deformity, inability to assume the supine position or extend the neck, rapidly progressive Grade II inhalation injury with airway edema, two failed attempts at direct laryngoscopy, progressive hypoxemia, and the need to perform emergency awake open tracheostomy while preserving spontaneous ventilation in a fully sitting position of approximately 90°. In the focused literature reviewed, we did not identify a closely comparable published case combining all of these features.

Awake fiberoptic intubation was considered in our patient, and the necessary equipment was available. However, after two unsuccessful direct laryngoscopy attempts, airway edema was progressing rapidly and oxygen saturation continued to decline. Under these circumstances, additional attempts at tracheal intubation were considered likely to delay definitive airway control and increase the risk of severe hypoxemia or complete airway obstruction. The team, therefore, proceeded directly to awake tracheostomy while spontaneous ventilation was still preserved. This decision reflected the rapidly evolving clinical situation rather than unavailability of fiberoptic equipment.

### 4.4. Clinical Implications

This case highlights the importance of individualized airway assessment and the need to avoid rigid adherence to standard positioning protocols when significant anatomical limitations are present. Airway management strategies should be tailored to each patient’s anatomical characteristics rather than forcing patients into conventional positions that may be harmful, ineffective, or delay definitive airway control.

Multidisciplinary collaboration among intensivists, anesthesiologists, and surgeons is essential in complex airway situations, facilitating timely decision‐making and selection of the most appropriate airway management technique before further clinical deterioration occurs. In emergency settings within the intensive care unit, airway management decisions often need to be made rapidly at the bedside, requiring both experience and proactive judgment from the treating team. Clinicians caring for burn patients should maintain a low threshold for airway intervention when inhalation injury is suspected, particularly in patients with preexisting anatomical abnormalities such as ankylosing spondylitis or cervical spine deformities.

Readiness to employ surgical airway techniques when conventional approaches are unsuccessful, together with appropriate training and system preparedness, may contribute to more effective airway control in similar high‐risk situations.

## 5. Conclusion

Awake tracheostomy in the sitting position may be an effective option for securing the airway in selected patients with a difficult airway when endotracheal intubation is unsuccessful and conventional supine positioning is not feasible because of severe anatomical limitations. In patients with rapidly progressive airway compromise, preservation of spontaneous ventilation while establishing a definitive surgical airway may be particularly important.

This case highlights the importance of early airway assessment, individualized airway management planning, and readiness to transition promptly to a surgical airway when conventional approaches are unsuccessful or are considered likely to delay definitive airway control.

## Author Contributions

Van Quynh Nguyen: conceptualization, manuscript drafting, and literature review.

Quang Thao Le: patient management, data collection, and manuscript revision.

Thi Van Nguyen: radiological interpretation, image acquisition, and manuscript revision.

## Funding

The authors received no specific funding for this work.

## Ethics Statement

Institutional ethical approval was not required for this single‐patient case report.

## Consent

Written informed consent was obtained from the patient for publication of the clinical details and accompanying images included in this case report.

## Conflicts of Interest

The authors declare no conflicts of interest.

## Supporting Information

Additional supporting information can be found online in the Supporting Information section.

## Supporting information


**Supporting Information** CARE_Checklist_Sitting_Tracheostomy.

## Data Availability

The data supporting the findings of this case report are available from the corresponding author upon reasonable request.
