# Supplementary material for: Awake Tracheostomy in the Sitting Position for a Predicted Difficult Airway in Ankylosing Spondylitis With Inhalation Injury: A Case Report
Source: Case Rep Anesthesiol. 2026 Sep 20;2026:7792815. doi: 10.1155/cria/7792815 (PMC13591014; doi:10.1155/cria/7792815)
Supplement: Supplementary file 1 [file CRIA-2026-7792815-s001.docx]

**CARE Checklist**

| **Topic** | **Item No.** | **Checklist Item Description** | **Reported on Page No.** |
| --- | --- | --- | --- |
| Title | 1 | The words “case report” are included in the title. | 2 |
| Key Words | 2 | Key words identify the main elements of this case. | 3 |
| Abstract | 3a | Introduction: What is unique about this case and what does it add to the medical literature? | 3 |
| Abstract | 3b | Main symptoms and/or important clinical findings. | 3 |
| Abstract | 3c | Main diagnoses, interventions, and outcomes. | 3 |
| Abstract | 3d | Conclusion and main lessons learned from this case. | 3 |
| Introduction | 4 | Brief background summary and relevant literature. | 4 |
| Patient Information | 5a | Demographic information. | 4 |
| Patient Information | 5b | Main symptoms and chief complaints. | 4–5 |
| Patient Information | 5c | Medical history. | 5 |
| Clinical Findings | 6 | Significant physical examination findings. | 4–5 |
| Timeline | 7 | Historical and current information organized chronologically. | 4–5 |
| Diagnostic Assessment | 8a | Diagnostic methods and findings. | 5 |
| Diagnostic Assessment | 8b | Diagnostic challenges. | 5–6 |
| Therapeutic Intervention | 9a | Types of intervention. | 6–7 |
| Therapeutic Intervention | 9b | Administration of intervention. | 6–7 |
| Follow-up and Outcomes | 10a | Clinician-assessed outcomes. | 7 |
| Follow-up and Outcomes | 10b | Important follow-up results and outcomes. | 7 |
| Discussion | 11a | Strengths and limitations of the approach. | 7–10 |
| Discussion | 11b | Relevant medical literature. | 7–10 |
| Discussion | 11c | Scientific rationale and conclusions. | 7–10 |
| Patient Perspective | 12 | Patient perspective or experience. | Not reported |
| Informed Consent | 13 | Written informed consent was obtained from the patient for publication of this case report and accompanying images. | 12 |
